# Identification and Validation of Marketing Weight-Related SNP Markers Using SLAF Sequencing in Male Yangzhou Geese

**DOI:** 10.3390/genes12081203

**Published:** 2021-08-03

**Authors:** Sherif Melak, Qin Wang, Ye Tian, Wei Wei, Lifan Zhang, Ahmed Elbeltagy, Jie Chen

**Affiliations:** 1 Laboratory of Molecular Genetics and Animal Breeding, College of Animal Science and Technology, Nanjing Agricultural University, Nanjing 210095, China; melakshaa@yahoo.com (S.M.); 2016105002@njau.edu.cn (Q.W.); Hollytianye@163.com (Y.T.); wei-wei-4213@njau.edu.cn (W.W.); lifanzhang@njau.edu.cn (L.Z.); 2 Laboratory of Genetics, Animal Production Research Institute, Agricultural Research Center, Giza 12618, Egypt; ahmed.elbeltagui@gmail.com

**Keywords:** SLAF-seq, BSA, SNPs, marketing weight, Yangzhou geese

## Abstract

Growth performance is a complex economic trait for avian production. The swan goose *(Anser cygnoides*) has never been exploited genetically like chickens or other waterfowl species such as ducks. Traditional phenotypic selection is still the main method for genetic improvement of geese body weight. In this study, specific locus amplified fragment sequencing (SLAF-seq) with bulked segregant analysis (BSA) was conducted for discovering and genotyping single nucleotide polymorphisms (SNPs) associated with marketing weight trait in male geese. A total of 149,045 SNPs were obtained from 427,093 SLAF tags with an average sequencing depth of 44.97-fold and a Q30 value of 93.26%. After SNPs’ filtering, a total of 12,917 SNPs were included in the study. The 31 highest significant SNPs—which had different allelic frequencies—were further validated by individual-based AS-PCR genotyping in two populations. The association between 10 novel SNPs and the marketing weight of male geese was confirmed. The 10 significant SNPs were involved in linear regression model analysis, which confirmed single-SNP associations and revealed three types of SNP networks for marketing weight. The 10 significant SNPs were located within or close to 10 novel genes, which were identified. The qPCR analysis showed significant difference between genotypes of each SNP in seven genes. Developed SLAF-seq and identified genes will enrich growth performance studies, promoting molecular breeding applications to boost the marketing weight of Chinese geese.

## 1. Introduction

Growth performance is the most important economic factor in the poultry industry. In poultry breeding, males are usually selected for growth and meat production, while females are selected for reproduction [1]. Chinese geese are very prolific and are considered the best egg laying breed, but are listed in the lightweight class [2]. A synthetic breed, Yangzhou geese, is a main breed in China, approved as the “first national geese breed” by the National Examination and Approval Committee of Domestic Animal and Poultry Breeds in 2006 [3]. It is a medium body-sized dual-purpose breed for meat and egg production [4]. The average body weight for Yangzhou goslings at nine weeks of age is 3.26 kg [5].

Genetic variations at candidate genes—associated with economic traits such as growth and meat production—have stimulated research in marker-aided selection (MAS) and evolutionary relationships studies [6]. DNA-based molecular markers techniques are costly, time-consuming, and some of them lack reproducibility of genotyping results [7]. Nowadays, single nucleotide polymorphisms (SNPs) are the most adopted and stable technique for studying genomic polymorphism [8]. They provide an assessable association with economic traits via linkage map construction and are utilizable for marker-aided selection strategies [9]. Thousands of markers can be discovered rapidly and efficiently by analyzing sequencing data. Recently, specific locus amplified fragment sequencing (SLAF-seq) has been developed to identify SNPs using the rapid development of high-throughput sequencing and application of next generation sequencing technologies [10]. Genome-wide association studies (GWASs) have been used to discover associated SNPs and identify genes associated with complex economic traits in avian species such as chicken egg production, chicken body weight, chicken meat quality and body composition, and geese egg laying [11,12,13,14,15,16].

SLAF-seq is a technique that uses an enhanced reduced-representation genome sequencing method for discovering de novo SNPs that can be used for large-scale genotyping [10]. SLAF-seq has several advantages that enable it to be an efficient, accurate, fast, and cost-effective method to discover de novo SNP markers in species. It can be typed among populations and can be applied for species with or without a reference genome [10,17]. The technique that involves genotyping of only one pair of pooled DNA samples, collected from two groups of individuals with opposite extreme phenotypes to identify markers associated with target genes/QTLs and influence the interested trait, is called bulked segregant analysis (BSA) [18]. It is a cost-effective method and is less sensitive to occasional phenotyping mistakes [19]. GWAS using SLAF-seq was conducted to identify significant SNPs related to growth traits in Jinghai Yellow, Chinese Yancheng, and Wenshang Barred chicken breeds [20,21,22].

According to the lack of polymorphism information and incomplete geese reference genome sequence, GWA study on the body weight of male goslings at nine weeks of age (marketing weight) was conducted for the first time using a combination of SLAF-seq and BSA (SLAF–BSA) techniques followed by a replication association study to detect and identify significant SNPs related to marketing weight (MW) in Yangzhou goslings. Identification of genes related to variations in MW of male goslings could provide new insight into the genetic basis of this trait and utilize it in designing efficient early selection strategies in geese breeding programs to promote the production of geese with high marketing weight and earlier marketing age.

## 2. Materials and Methods

### 2.1. Ethics Statement

All experimental protocols and animal care were reviewed and approved by Animal Care of Nanjing Agricultural University. The protocols were conducted in compliance with the Administration of Affairs Concerning Experimental Animals Regulations (China, 1988). All efforts were made during blood collection and geese slaughtering to minimize any discomfort.

### 2.2. Samples and Phenotypic Measurements

A total of 167 male Yangzhou goslings (*Anser cygnoides*) of the first population (P1), belonging to Jiangsu Lihua Animal Husbandry Co. Ltd. commercial farm (Changzhou, China) were randomly selected and utilized for SNPs’ detection in the present study. Rice grains were used to feed goslings ad libitum and supplemented with green grass or water plants, whenever available. Goslings are fed on a diet containing 12 MJ/kg of energy and 18% of protein before three weeks of age and on a diet containing 10 MJ/kg of energy and 12% of protein after three weeks of age. Goslings were housed in a semi-enclosed building and raised under the same conditions according to the farm’s standard practice with stock density of 4.5 goslings/m^2^. Goslings were released into the barn yard during the daytime and fed at this time. Goslings were exposed to natural temperature and lighting throughout the study. Individual records of goslings’ body weight at nine weeks of age (marketing weight) were obtained from the farm. Marketing weight (MW) data were checked for normality before conducting any further analysis. Twenty individuals from each of the lowest and the highest male goslings groups of the P1 were used in the SLAF study based on best linear unbiased prediction (BLUP) of individual estimated breeding values (EBV) for MW trait, which had been assessed using full and half sibs’ information by the lme4 package of R software [23] according to [24].

### 2.3. Preparation and Construction of SLAF Library

Blood samples (2 mL) were collected from wing veins of 167 male goslings at nine weeks of age and immediately transferred into 5 mL tubes containing acid citrate dextrose and stored at −20 °C pending the DNA extraction. The conventional phenol/chloroform method was used to extract genomic DNA. The concentration and purity of DNA for each individual sample was assessed using the Thermo Scientific NANODROP2000 spectrophotometer (Thermo Fisher Scientific Inc., Waltham, MA USA). DNA concentration was adjusted to 100 ng/μL for each sample. Twenty samples from each of the low estimated breeding values (LEBV) and high estimated breeding values (HEBV) groups were used to prepare two DNA pools by mixing an equal amount of genomic DNA. The SLAF-seq technique was used to develop and obtain molecular markers across the whole genome data of the two goslings groups by Beijing Biomarker Biotechnology Co., Ltd. (http://www.biomarker.com.cn/ accessed on 27 April 2018) [10]. Briefly, the geese (GOOSE) genome was used as the reference genome for electronic enzyme digestion prediction based on the actual size of goose genome and GC content. The genomic DNA of each sample was digested separately. The restriction enzymes (RsaI and HaeIII) were used to digest genomic DNA. After sequencing, the libraries were qualified with Illumina HiSeq TM2500 (Illumina, Inc., San Diego, CA, USA). In order to evaluate the accuracy of the enzyme digestion experiments, Japanese rice (Oryza sativa ssp. *japonica*) was used as a control for sequencing (http://rapdb.dna.affrc.go.jp/ accessed on 27 April 2018).

### 2.4. Sequencing Analysis and Detection of MW-Related SNPs 

A total of 384,079 SLAF tags with an average sequencing depth of 25-fold were developed for each sample. In order to ensure the quality of the project analysis, a reading length of 100 bp × 2 was used as the subsequent data evaluation and analysis. The short oligonucleotide alignment program (SOAP) was used to compare the sequencing reads of control with its reference genome [25]. For the development of SNP markers, the Burrows–Wheeler alignment tool (BWA) was used to align the sequencing reads with reference genome [26]. Two methods of the sequence alignment/map format (SAMtools) [27] and the genome analysis toolkit (GATK) [28] were used to develop the SNPs list. The SNP marker intersection obtained by both methods was used as the final reliable SNP marker dataset, thus a total of 149,045 SNPs were obtained. LEBV and HEBV SlAF-seq libraries were submitted to NCBI’s Sequence Read Archive with the accession numbers SRR14113841 and SRR14113842, respectively.

### 2.5. Quality Control of SLAF Tags

The sequencing quality value (Q) was used to assess the quality of raw SLAF reads. The corresponding formula of base sequence error rate *p* and sequence quality value is (Q-score = −10 × log10 *p*). If the sequence accuracy is 99.9%, the quality value for the base should be 30. Both the Euclidean distance (ED) and SNP index methods were then used to filter data for either LEBV or HEBV cohorts according to [29,30]. Genotypes’ differences in the SNP loci between the both pools were used to calculate the depth of each base and, therefore, the values of ED for each locus. The following equation was used to calculate ED at each SNP location:

(1) $$\mathrm{ED}\;=\sqrt{{\left(A_{\mathrm{HEBV}}-A_{\mathrm{LEBV}}\right)}^{2\;}+{\left(T_{\mathrm{HEBV}}-T_{\mathrm{LEBV}}\right)}^{2}+{\left(G_{\mathrm{HEBV}}-G_{\mathrm{LEBV})}\right)}^{2}+{\left(C_{\mathrm{HEBV}}-C_{\mathrm{LEBV}}\right)}^{2}}$$

where, A_HEBV_, C_HEBV_, T_HEBV_, and G_HEBV_ are the depth of A, C, T, and G bases on a site in HEBV bulk, respectively. A_LEBV_, C_LEBV_, T_LEBV_, and G_LEBV_ are the depth of A, C, T, and G bases on a site in LEBV bulk, respectively. The ∆(SNP index) formula used to calculate the differences of genotypic frequency between LEBV and HEBV bulks was as follows:

SNP index of LEBV = M_LEBV_/(P_LEBV_ + M_LEBV_) (2)

SNP index of HEBV = M_HEBV_/(P_HEBV_ + M_HEBV_) (3)

∆(SNP index) = SNP index of HEBV-SNP index of LEBV(4)

where M and P stands for HEBV and LEBV, respectively. HEBV and LEBV denote the genotype from high and low estimated breeding values pools, respectively. M_HEBV_ and P_HEBV_ are the depth of the HEBV population derived from M and P, respectively. M_LEBV_ and P_LEBV_ are the depth of the LEBV population derived from M and P, respectively. 

All SNPs that met the condition ED ≥ 0.7, SNP index ≥ 0.5, and Q ≥ 30 were involved in the present analysis. After data filtering, 12,917 SNPs were used for further analysis. Allele frequency differences for each SNP were used to compare between LEBV and HEBV pools. SNPs with a highly significant difference in the allelic distribution of both pools were selected as candidate loci for further verification in the population.

### 2.6. Replication Association Study

For the replication study, blood and MW of 114 male Yangzhou goslings were collected from individuals of the second population (P2) derived from the same farm. MW data were checked for normality before conducting any further analysis. P2 was kept under the same management system of P1. Blood samples (2 mL) were collected, reserved, and DNA was extracted and its quality was assessed with the same protocol described in Section 2.3.

### 2.7. Genotyping of Female Goslings of First and Second Populations 

In order to investigate if the candidate SNPs are gender-related, blood samples and MW of 323 and 345 female Yangzhou goslings belonging to the same farm were collected from individuals of the P1 and P2, respectively. Firstly, the discovered SNPs that showed significantly MW-related SNPs between HEBV and LEBV male goslings groups were genotyped using AS-PCR method in 24 individuals from each of the lowest and the highest female gosling groups of P1 based on EBV. The SNPs that showed a significant difference between female goslings of LEBV and HEBV were secondly genotyped using the AS-PCR method in 323 and 345 female goslings of P1 and P2, respectively. Female goslings of P1 and P2 were kept under the same management system of male goslings of P1 and P2. Blood samples were collected and reserved, and DNA was extracted and its quality was assessed with the same protocol described in Section 2.3.

### 2.8. Verification of MW-Related SNP Genotypes

Based on SLAF-BSA analysis and Chi square test, the 31 highest significantly candidate SNPs of different allelic distribution were selected for male individual-based genotyping in the LEBV and HEBV cohorts of P1. A total of 13 SNPs that showed significantly different allele distribution for MW in the LEBV and HEBV cohorts were further verified in all male individuals of P1 and P2 using the AS-PCR genotyping method. The 13 SNPs were also genotyped using the AS-PCR method in the lowest and the highest female gosling groups of P1 and the significant SNPs were further genotyped in all female individuals of P1 and P2. Primer Premier 5 software (PREMIER Biosoft, Palo Alto, CA, USA) was used to design each pair of primers to amplify fragments based on GOOSE genomic DNA sequence (https://www.ncbi.nlm.nih.gov/ accessed on 30 June 2018). The primers for AS-PCR were designed according to [8,31]. An additional mismatch base pair was inserted at the third base from the 3′ end to improve the specificity of PCR amplification and reliable discrimination between the alleles. The SNPs, their primers, and PCR product length are shown in Table S1. Genotypes with two specific primers were performed in duplicates in a total of 20 μL reaction volumes containing 10 μL r-taq (Takara, Dalian, China), 1 μL from each of the forward and reverse primers (Tsingke, Nanjing, China), 1 μL DNA template, and 7 μL ddH_2_O. PCR amplification was carried out by preliminary denaturation at 94 °C for 5 min followed by 32 cycles of amplification (denaturing at 94 °C for 30 s, annealing at ATm °C for 30 s, and extension at 72 °C for 30 s) and a final extension at 72 °C for 7 min. All PCR products quantity were fractionated by agarose gel (2%) electrophoresis, visualized with gold view staining, and quantified with Tanon 3500 Gel Imaging system (Tanon Science and technology Co., Ltd., Shanghai, China).

### 2.9. Quantitative Real-Time PCR (RT-qPCR)

Seven male goslings from each of the HEBV and LEBV groups of P2 were selected—according to their genotypes—and slaughtered to obtain brain tissues (pre-experiments of gene expression were performed to select the suitable tissue). Tissues were collected and immediately frozen in liquid nitrogen pending the expression analysis. Total RNA was extracted from the brain tissues using TRIZOL Reagent (Invitrogen, Carlsbad, California, USA) according to the manufacturer’s protocol. RNA quality and quantity were assessed by NanoDrop spectrophotometer at 260/280 nm. The cDNA first strand was synthesized from 1 μg of purified total RNA using ProtoScript First Strand cDNA Synthesis kit (Takara, Dalian, China). The gene specific primers of the identified 10 candidate genes harboring MW-related SNPs were designed using Primer Premier 5 software to determine the expression levels of the 10 genes in geese brain tissue by qPCR. The primers (Table S2) were designed based on the geese genome sequence of these genes in the Genbank database (*Anser cygnoides*, *taxid:8845*). SYBR^®^ Green Master Mix (Vazyme, Nanjing, China) was used in a StepOne Plus Real-Time PCR system (Applied Biosystems, Foster City, CA, USA). The PCR reaction (20 µL) consisted of 1 µL cDNA, 0.4 µL from each primer (10 µmol), 0.4 µL ROX Reference Dye, 10 µL SYBR Green Master Mix, and 7.8 µL nuclease-free water. Amplification thermal conditions were as follows: pre-denaturation at 95 °C for 5 min, 40 amplification cycles (95 °C for 10 s and 60 °C for 30 s). Melting curve analysis was performed from 60 °C to 95 °C by reading plate every 0.1 °C. Each sample was analyzed three times. The house-keeping gene glyceraldehyde-3-phosphate dehydrogenase (*GAPDH*) was used as internal control.

### 2.10. Statistical, Bioinformatics, and Data Analysis

Function Shapiro test of R [23] was used to perform a Shapiro–Wilk test for goslings MW normality. In male and female goslings of both populations, the best linear unbiased predictions of individual estimated breeding values for MW trait were estimated using full and half sibs’ information by lme4 package of R software [23] according to [24]. The formula used to predict the BV is as follows: â_ijk_ = 1/2 (â_j_ + â_k_) + ŵ_ijk_, where â_ijk_ = the BV i^th^ progeny of parent j x parent k, â_j_ = the BV of parent j, â_k_ = the BV of parent k, and ŵ_ijk_ = the within-family Mendelian additive effect of the individual ijk. ŵ was calculated as ŵ_ijk_ = h^2^w (e_ijk_), where e_ijk_ = the residual from the linear mixed model associated with progeny from parent j × parent k, and h^2^w = the within-family heritability = 1/2 σ^2^A/σ^2^e. The allelic frequency variation of SLAF tags between LEBV and HEBV male groups were tested using contingency tables and chi-square statistics. Two methods, false discovery rate and Bonferroni correction, were used to obtain adjusted *p*-values and estimate the significance threshold level at 5% overall Type I error rate [32,33]. One-way analysis of variance (ANOVA) in the statistical language R was used to test the association between different genotypes and MW. Mean separation test of Duncan was used to compare between means [34]. PLINK software was used to estimate genetic and allelic frequencies [35]. All single SNP–trait associations that reached a significance level of *p* < 0.05 were included in further multiple marker analysis. Multiple-marker associations were analyzed with two quantitative trait modes (additive mode: PAa (PAA + Paa)/2) and dominant mode: PAa either PAA or Paa) by the linear regression procedure of SAS software using forward or backward stepwise comparison [36] according to [37]. All data were expressed as mean ± SE. Spearman rank correlation matrices were performed by SAS software using SNPs’ genotype values to design heat map. According to the 10 significant SNPs found to be associated with MW in male gosling of P1 and P2, a BLAST analysis against the *NCBI* public database (https://blast.ncbi.nlm.nih.gov/Blast.cgi accessed on 20 March 2020) using the SLAF-taqs of 100 bp sequence was performed to retrieve orthologous sequences. The geese sequences in the whole-genome shotgun contigs (*wgs*) database of *Anser cygnoides* (*taxid:8845*) were used for alignment. The 2^−ΔΔCT^ method was used to calculate relative quantification of gene expression. *GAPDH* was used as an internal control. Two tailed t-tests were used to analyze mRNA expression variation between different genotypes in each tested SNP.

## 3. Results

### 3.1. Analysis of Goslings’ Marketing Weight 

**Figure 1:**
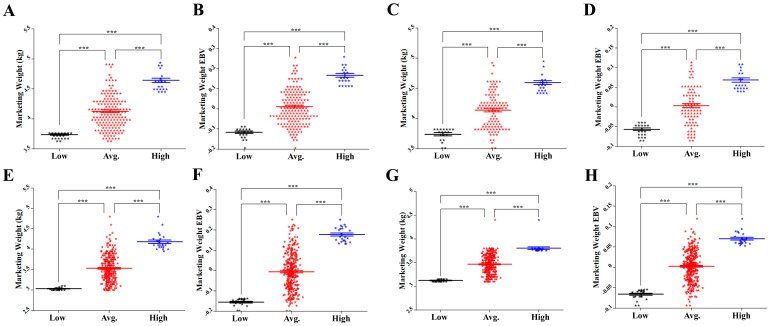
Average marketing weight (MW) and estimated breeding values (EBVs) for males and females from P1 and P2: (**A**) MW and (**B**) EBV of P1 males, (**C**) MW and (**D**) EBV of P2 males, (**E**) MW and (**F**) EBV of P1 females, and (**G**) MW and (**H**) EBV of P2 females between high, average, and low EBV groups. The X axis represents low, average, and high groups of males and females from P1 and P2. The Y axis represents MW (kg) or EBV. The error bars represent the standard error of mean. * *p* < 0.05, ** *p* < 0.01 and *** *p* < 0.001.

Gander had a higher marketing weight (MW) than goose. The average MWs were 4.12 and 4.09 kg for males and 3.54 and 3.47 kg for females in the first (P1) and second (P2) populations, respectively. The average estimated breeding values (EBVs) of males (0.01 and 0.003) were approximately equal to those of females (−0.006 and 0.002) in both populations. The heritability of MW was 0.29 and the environmental proportion of total variance was 0.71. Figure 1 shows MW (kg) and EBVs for the MW trait of each individual, divided into low, average, and high groups in the male and female goslings of P1 and P2.

### 3.2. SLAF Sequencing

Table 1 shows the statistics of sequencing data for each sample including the number of reads, quality value (Q30), and GC content. A total of 5.4 Gb of sequencing data were generated by SLAF sequencing containing more than 20 M paired-end mapped reads representing 95.39% of the total reads. Through bioinformatics analysis, 427,093 SLAF tags were obtained with an average sequence depth of 44.97-fold. A total of 149,045 SNPs were obtained from polymorphism of 84,594 SLAF tags. The average Q30 sequence was 93.26% with an average GC content of 42.88%. After filtering by SNP index and Euclidean distance (ED), 12,917 SNPs were included in downstream analysis. The ratio of transition/transversion (ti/tv) was 2.33, where 69.96% was transition and 30.04% was transversion. For the transitions category, the percentage of A–G substitution (35.54%) roughly equals that of C–T substitution (34.42%), while the percentages of G–T (7.58%), A–C (8.09%), A–T (7.07%), and C–G (7.30%) substitutions are almost equal in transversions.

### 3.3. Discovering of Goslings MW-Related SNPs

For all 12,917 SNPs detected by SLAF sequencing data, an independent Chi square test was used to estimate allele frequency differences between males of high (HEBV) and low (LEBV) estimated breeding value cohorts. A total of 3145 SNPs showed a significant effect in the Chi square test (*p* < 0.05), 382 SNPs reached a 5% false discovery rate (FDR), and only 68 SNPs reached 5% Bonferroni correction. The 31 highest significant SNPs (*p* < 2.26 × 10^−6^–9.22 × 10^−33^) were selected as candidate MW-related SNPs (Table S1). Allele-specific polymerase chain reaction (AS-PCR) was used to genotype forty male individuals; as twenty from each of the HEBV and LEBV cohorts. Thirteen out of 31 SNPs showed significant (*p* < 0.04–8.44 × 10^−7^) allele frequency differences between HEBV and LEBV male groups (Figure S1). Details on the significant SNPs including their genomic positions and *p*-values for males of P1 are summarized in Table 2.

### 3.4. Verification of MW-Related SNPs in Male Goslings

**Figure 2:**
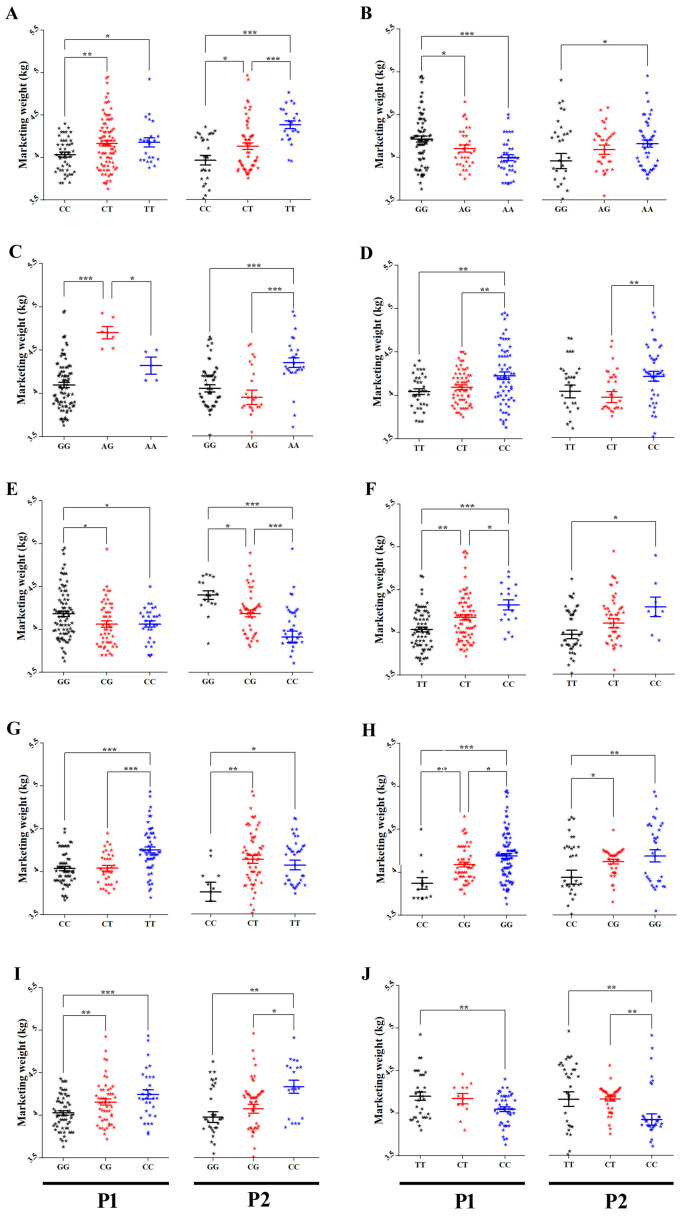
(**A**) Average marketing weights (kg) of each genotype of Record_1102, (**B**) Record_1111, (**C**) Record_2315, (**D**) Record_1009, (**E**) Record_1056, (**F**) Record_1115, (**G**) Record_7099, (**H**) Record_7097, (**I**) Record_8964, and (**J**) Record_396 SNPs in males of the P1 and P2, respectively. The X axis represents different genotypes groups of each SNP in males of the P1 and P2. The Y axis represents MW (kg). The error bars represent the standard error of mean. * *p* < 0.05, ** *p* < 0.01 and *** *p* < 0.001.

Using the AS-PCR method, a total of 13 SNPs that showed a significant influence on MW of HEBV and LEBV male cohorts were selected as candidate SNPs to be associated with MW in 167 males of P1. Ten out of the 13 SNPs reached a 5% Bonferroni distribution over five different chromosomes. Duncan separation test showed that goslings with TT genotype of SNPs Record_1102, Record_7099, and Record_396 loci were of higher MW than those with CC genotype. For the heterozygous, there was a significant difference in MW between goslings with CT and CC genotypes in Record_1102 and goslings with TT genotype in Record_7099 loci. At loci of Record_1056 and Record_7097, the GG was linked with higher MW than the genotype CC. The CC genotype at Record_8964 locus was related to higher MW than the GG genotype. There was a significant difference between CG and GG genotypes in Record_1056 and Record_8964 loci. There was a significant difference in MW between goslings with CG and both homozygous in Record_7097 locus. Individuals having CC genotype showed higher MW than the individuals having TT genotype in Record_1009 and Record_1115 loci. There was a significant difference in MW between CT and CC genotypes in Record_1009 locus. There was a significant difference between CT and both of homozygous genotypes in Record_1115 locus. For Record_1111, the GG genotype was associated with higher MW than the AG and AA genotypes. Heterozygous AG genotype was related to higher MW than both homozygous genotypes at Record_2315 locus. Figure 2 shows the MW of each genotype of the 10 significant SNPs in P1 males.

### 3.5. Replication Association Analysis for Male Goslings

In order to validate the significance of the 10 SNPs that were shown to be associated with MW of males from P1, 114 individuals of males from P2 were genotyped using AS-PCR. The AS-PCR genotypes association analysis of the individuals from P2 confirmed the results obtained from the genotypes analysis of P1. Allelic and genotypic frequencies for males of both populations are shown in Figure S2. Goslings with TT genotype of Record_1102 locus had a significantly higher MW than those with CC and CT genotypes. Goslings with TT genotype of Record_396 locus had a significantly higher MW than those with CC genotype. Goslings with CT genotype of both SNPs loci had a significantly higher MW than those with CC genotype. Goslings with GG genotype in Record_1056 locus showed a significantly higher MW in comparison with those with CC and CG genotypes and goslings with CG genotype had also a significantly higher MW than those with CC genotype. In contrast, goslings with CC genotype in Record_8964 locus showed a significantly higher MW in comparison with those with GG and CG genotypes. Individuals with GG and CG genotypes of Record_7097 locus had a significantly higher MW than those with CC genotype. The homozygous CC genotype of Record_1009 locus had a significantly higher MW than CT genotype. The CC genotype of Record_1115 locus had a significantly higher MW than TT genotype. Goslings with TT and CT genotypes of Record_7099 locus had a significantly higher MW than those with CC genotype. Goslings with AA genotype of Record_1111 locus had a significantly higher MW than those with the GG genotype. Furthermore, goslings with AA genotype of Record_2315 locus had a significantly higher MW than those with AG and GG genotypes. Figure 2 shows the MW of each genotype of the 10 significant SNPs in P2 males.

### 3.6. Linear Regression Model and SNP Networks Analysis of Male Goslings Marketing Weight

**Figure 3:**
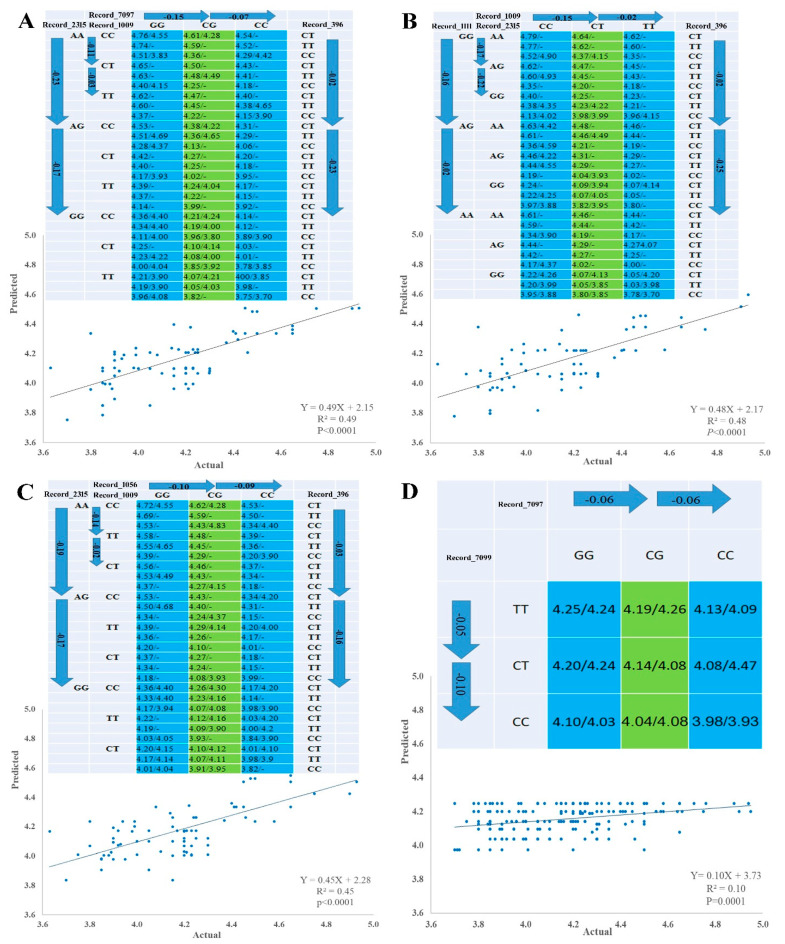
Genetic networks with multiple SNPs for marketing weight of male goslings. (**A**) Four SNP-networks for SNPs Record_2315, Record_1009, Record_7097 and Record_396 (SNP-network 1), (**B**) Four SNP-networks for SNPs Record_1111, Record_2315, Record_1009 and Record_396 (SNP-network 2), (**C**) Four SNP-networks for SNPs Record_2315, Record_1009, Record_1056 and Record_396 (SNP-network 3) and (**D**) Two SNP-networks for SNPs Record_7099 and Record_7097 (SNP-network 8). The X and Y axes represent actual and predicted MW (kg), respectively.

**Figure 4:**
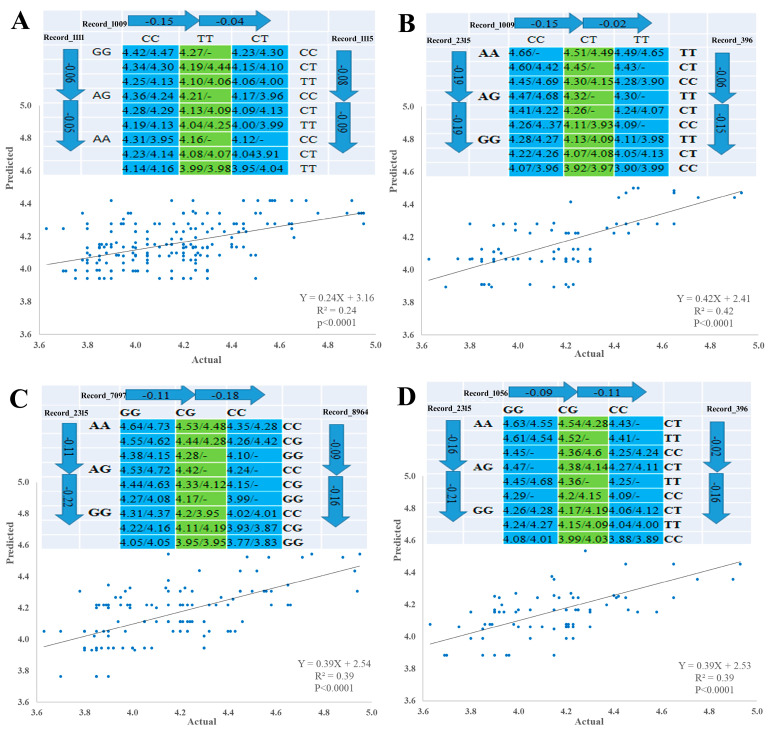
Genetic networks with multiple SNPs for marketing weight of male goslings. (**A**) Three SNP-networks for SNPs Record_1111, Record_1009 and Record_1115 (SNP-network 4). (**B**) Three SNP-networks for SNPs Record_2315, Record_1009 and Record_396 (SNP-network 5), (**C**) Three SNP-networks for SNPs Record_2315, Record_7097 and Record_8964 (SNP-network 6), and (**D**) Three SNP-networks for SNPs Record_2315, Record_1056 and Record_396 (SNP-network 7). The X and Y axes represent actual and predicted MW (kg), respectively.

Pairwise comparison test with either forward or backward methods was used to identify SNP–SNP combinations using linear regression model according to [37]. The 10 significant SNPs were involved in multiple regression model analysis, which verified single-SNP associations and revealed three types of SNP networks for MW of male goslings. The three types of SNPs networks are as follows: four SNP-networks; three SNP networks and two SNP networks (Figure 3 and Figure 4). All SNPs had an additive mode except Record_2315 and Record_7099, which a had dominant mode, and Record_1009, which had an over dominant mode, regarding single SNP association with MW. For the three types of SNP networks, all SNP networks showed additive–dominant combinations between SNPs. The substitution of GG–AA homozygotes reduced MW by 110 (SNP network 4) to 180 g (SNP network 2) at Record_1111 locus. For Record_2315 locus, the substitution of AA–GG homozygotes decreased MW by 330 (SNP network 6) to 400 g (SNP network 1). Substitution of CC–TT homozygotes at Record_1009 locus resulted in a decrease of MW by 140 (SNP-network 1) to 170 g (SNP-network 2) and at Record_1115 locus (SNP network 4). The substitution of GG–CC homozygotes at Record_1056 locus descended MW by 190–200 g. The MW increased by 150 g (SNP network 8) owing to the substitution of CC–TT at Record_7099 locus, while this increase was approximately 160 (SNP network 3 and 7) to 250 g (SNP network 2) at Record_396 locus owing to the substitution. The substitution of GG–CC resulted in a weight gain of 120 (SNP network 8) to 290 g (SNP network 6) at Record_7097 locus. On the contrary, this substitution at Record_8964 locus resulted in a weight reduction of 250 g (SNP network 6).

### 3.7. Additive, Dominance, and Recessive Effects of Significant SNPs 

The genotypic effects of the 10 significant SNPs of males in both populations were further divided into additive, dominant, and recessive effects. In P1, the values ranged between −0.118–0.265, −0.214–(−0.058), and −0.453–(−0.073) for additive, dominant, and recessive effects, respectively. For P2, the values ranged between −0.198–0.030, −0.430–0.104, and −0.247–(−0.006) for additive, dominant, and recessive effects, respectively. In general, all tested SNPs in the present study showed significant effects of one or more genotypes in P1 or P2. Additive, dominance, and recessive values of males in both populations are shown in Table 3.

### 3.8. Correlations between MW and SNPs’ Genotypes

**Figure 5:**
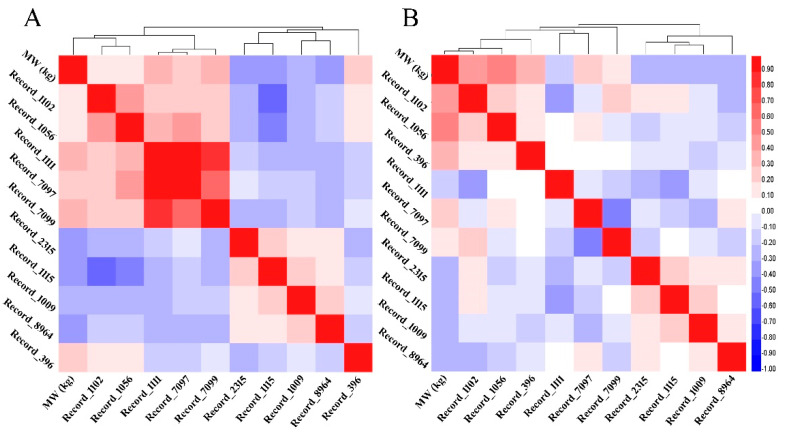
(**A**) Heat map with clustering analyses showing the pairwise Spearman correlations between all SNPs and marketing weight of males from the first and (**B**) second populations.

For more confirmation on SNP networks, two correlation matrixes based on coefficients of Spearman rank correlation were performed using SNPs’ genotype values. All SNPs of males from P1 and P2 showed a significant correlation with MW and, at the same time, most of them showed a significant correlation with themselves. The highest correlation coefficients were found between MW and SNPs of Record_1111, Record_7099, and Record_396 loci for P1 and SNPs Record_1056, Record_1102, and Record_396 loci for P2. The correlation coefficients between SNPs ranged between medium to unity in P1 and low to medium in P2. First and second populations heat map of all pairwise correlation between SNPs were constructed based on both correlation matrixes and visualized in Figure 5 with their clustering analyses.

### 3.9. Verification of MW-Related SNPs in Female Goslings

**Figure 6:**
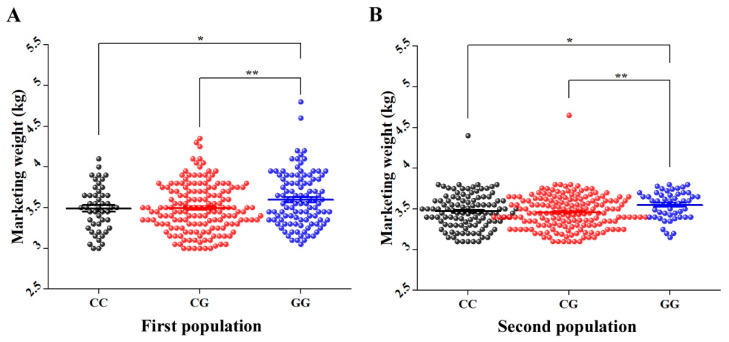
Average MW (kg) of each genotype of SNP Record_1056 in females of (**A**) first population and (**B**) second population. The X axis represents different genotypes groups of Record_1056 in females of P1 and P2. The Y axis represents MW (kg). The error bars represent the standard error of mean. * *p* < 0.05 and ** *p* < 0.01.

In order to investigate if the discovered SNPs are gender-related, the 13 significantly MW-related SNPs in HEBV and LEBV of male cohorts from P1 were used to genotype HEBV and LEBV of female cohorts from the same population by the AS-PCR method. The results showed that only 5 out of 13 SNPs showed significant allele frequency differences between HEBV and LEBV of female groups from P1 (Figure S1). Details on the significant SNPs including their genomic positions and *p*-values for females from P1 are summarized in Table 2. The five significantly associated SNPs of LEBV and HEBV female cohorts were further selected for individuals genotyping of 323 and 345 females of P1 and P2, respectively. The results showed a strong significant effect (*p* < 0.05) for only Record_1056. Goslings with GG genotype showed significantly higher MW than those with CC and CG genotypes in both populations (Figure 6).

### 3.10. Annotation of Genes Harboring SNPs Associated with Goslings MW

According to the 10 significant SNPs that were found to be associated with MW of males from P1 and P2, a BLAST analysis against the *NCBI* public database (https://blast.ncbi.nlm.nih.gov/Blast.cgi accessed on 20 March 2020) using the SLAF-taqs of 100 bp sequence was performed to retrieve orthologous sequences. The geese sequences in the whole-genome shotgun contigs (*wgs*) database of *Anser cygnoides* (*taxid:8845*) were used for alignment. The 10 significant SNPs were found to be located within or close to 10 genes that were concluded to be significantly associated with MW of male goslings. In general, the 10 SNPs are distributed in five different chromosomes including five SNPs (Record_1102, Record_1111, Record_1009, Record_1056, and Record_1115) on KZ155846.1, one SNP (Record_2315) on KZ155852.1, two SNPs (Record_7099 and Record_7097) on KZ155908.1, one SNP (Record_8964) on KZ155945.1, and one SNP (Record_396) on KZ155843.1 chromosomes. As shown in Table 4, SNP Record_1102 is located on intron four of H6 family homeobox 1 (*HMX1*) gene. SNP Record_1111 is located at 29.5 Kb upstream region of uncharacterized LOC106034756 gene. SNP Record_2315 is located at 10 Kb upstream region of LRR binding FLII interacting protein 1 (*LRRFIP1*) gene. SNP Record_1009 is located at 14.5 Kb upstream region of uncharacterized protein K02A2.6-like (*LOC106035299*) gene. SNP Record_1056 is located on intron one of protein phosphatase 2 regulatory subunit B γ (*PPP2R2C*) gene. SNP Record_1115 is located on intron one of uncharacterized LOC106034755 gene. SNP Record_7099 and Record_7097 are located at 3.3 Kb and 3 Kb, respectively, downstream region of ubiquitin C-terminal hydrolase L1 (*UCHL1*) gene. SNP Record_8964 is located at 28.3 Kb downstream region and 41.2 Kb upstream region of uncharacterized LOC106034143 and platelet derived growth factor D (*PDGFD*) genes. SNP Record_396 is located at 24.6 Kb upstream region of extracellular leucine-rich repeat and fibronectin type III domain containing 1 (*ELFN1*) gene.

### 3.11. Relative Gene Expression 

**Figure 7:**
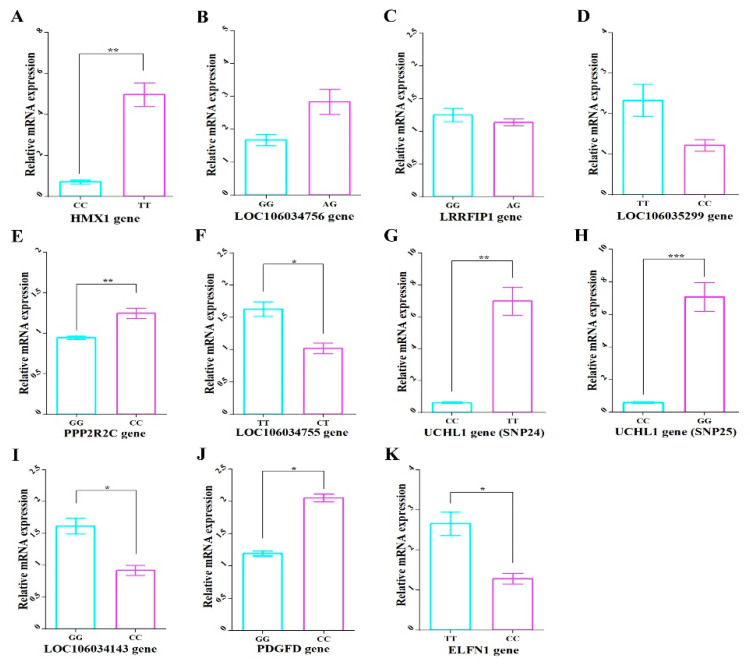
The mRNA expression levels of the 10 genes harboring significant SNPs of the different genotypes of each SNP. The mRNA of (**A**) *HMX1*, (**B**) *LOC106034756*, (**C**) *LRRFIP1*, (**D**) *LOC106035299*, (**E**) *PPP2R2C*, (**F**) *LOC106034755,* (**G**) *UCHL1* (Record_7099)*,* (**H**) *UCHL1* (Record_7097) (**I**) *LOC106034143,* (**J**) *PDGFD,* and (**K**) *ELFN1 genes.* The X axis represents different genotype groups of each gene in males of P2. The Y axis represents relative mRNA expression. The error bars represent the standard error of the mean. * *p* < 0.05, ** *p* < 0.01 and *** *p* < 0.001.

For further confirm that these genes are strongly related to MW, relative gene expression using qPCR was performed. The substitution of reference-alternative genotypes was shown to lead to upregulated mRNA expression levels in five genes (*HMX1, LOC106034756, PPP2R2C, UCHL1,* and *PDGFD*) and downregulated levels in five genes (*LRRFIP1, LOC106035299, LOC106034755, LOC106034143,* and *ELFN1*) (Figure 7). The mRNA expression levels in all genes showed significant differences (*p* < 0.05) between genotypes except *LOC106034756, LOC106035299,* and *LRRFIP1* genes. For *LOC106034756* and *LRRFIP1* genes, the alternative homozygous genotype is difficult to detect and there is no significant difference between reference homozygous and alternative heterozygous genotypes for MW in the second population (Figure 2).

## 4. Discussion

Goslings’ growth is a crucial factor for the whole life with a major impact on the efficiency of production and reproduction. Many factors influence body weight and growth performance of goslings; some are hereditary in origin (biological factors) and others are environmental factors. China dominates the global geese industry by producing approximately 4.8 million tons of meat, owning more than 90% of the global goose population [38]. Chinese geese have been kept for eggs and meat. They are relatively good egg layers. They actively forage and produce the least greasy meat of all but Pilgrim geese. However, Chinese geese are listed in the lightweight category [2]. Chinese geese are relatively small in size with an average mature body weight of 3.5–4.5 Ib for goose and 4.5–5.5 Ib for gander. Their average mature body weights are lower than the other global geese breeds such as Toulouse (10–13 for goose and 12–15 Ib for gander), Embden (10–13 for goose and 12–15 Ib for gander), and American Buff (9–12 for goose and 10–12 Ib for gander) [39]. In this study, the average marketing weight (MW) of Yangzhou goslings (3.50 kg for goose and 4.11 kg for gander) at nine weeks of age is higher than those previously reported for the same breed [5,40], but lower than the other global geese meat breeds such as Pomeranian (5.29), Landes (4.74), and Steinbacher (4.33) goose breeds at ten weeks of age [41]. Nowadays, the tissue composition of carcasses, body weight at slaughter, and meat quality are more important in the selection of breeds for meatiness.

Owing to the low average MW in Yangzhou geese, it was imperative to find a method for genetic improvement of growth, which can be utilized in selection programs. Achieving significant success using quantitative genetics tools depends on accurate data, a good management system, and unbiased distribution of ganders. Furthermore, it required several years to achieve the desired success. Genetic variations at candidate genes regarding economic traits have stimulated research interest [6]. GWA studies were used for rapidly and efficiently discovering thousands of associated SNPs and identifying genes for complex economic traits. SLAF-seq is the most efficient method of large-scale de novo SNP discovery that can be used in GWAS [10].

In this study, an economic and effective method of SLAF-seq with BSA techniques (SLAF-BSA) was employed for discovering and genotyping MW-related SNPs. A total of 427,093 SLAF-tags were obtained with an average sequencing depth of 44.97-fold. A total of 149,045 SNPs were obtained from polymorphism of 84,594 SLAF tags. After quality control by SNPs index and ED corrections, 12,917 SNPs were included in this study. Based on Chi square test, the 31 highest significant SNPs that reached 5% Bonferroni correction were selected as candidate MW-related SNPs of male goslings. Thirteen out of 31 SNPs showed significantly variation (*p* < 0.05) in allele frequency between males of HEBV and LEBV groups. The 13 SNPs were genotyped in 167 males of P1. Ten out of the 13 SNPs reached 5% Bonferroni distributing. A follow-up replication study in 114 males of P2 was conducted for further verification of the SNP markers’ impact on MW. The results verified the significant association of the 10 SNPs with MW. Only Record_1056 showed a significant (*p* < 0.05) association with MW for 323 and 345 females of P1 and P2, respectively. These results suggested that these SNPs are associated with MW in Yangzhou geese, and this effect might relate to gender. The ti/tv value is an important property for evolution of the DNA-sequence where transition has less impact than transversion in changing amino acids in the protein, despite its higher frequency in the genome [42]. The value of ti/tv ratio (2.33) mediated previously published drosophila (2.00) and human (4.00) values [43,44].

To the best of our knowledge, this is the first time the SLAF-BSA approach has been used to distinguish large-scale de novo SNPs related to MW in Yangzhou geese. Different ways have been used for discovering and genotyping SNPs in goose populations. Next generation sequencing was performed using reduced representation (RR) sequencing from a DNA pool to detect 2188 SNPs for Barnacle goose [45]. Using the candidate gene approach, two SNPs in exon two of the growth hormone gene were detected in Huoyan goose by genotyping 552 individuals using polymerase chain reaction [46]. Using restriction site associated DNA of two DNA pools, 139,013 SNPs related to egg laying in Yangzhou goose were discovered [16]. Moreover, two SNPs in exon one and one SNP in intron two of *SMAD* family member 9 gene were discovered in goose by the PCR-SSCP method [47]. Using reduced representation (RR) sequencing, 277,362 SNPs were detected for pink-footed goose [48]. In additional, one SNP in exon three of Myostatin gene was detected in Landes and Kielecka geese breeds [49]. Recently, a total of 26 SNPs and 14 annotated genes significantly associated with quality traits and egg production were identified in Sichuan white geese by GWAS [50].

Regarding the 10 significant SNPs of male goslings in both populations—which were detected in the current study—five SNPs (Record_1102, Record_1111, Record_1009, Record_1056, and Record_1115 loci) are distributed in the ~4 Mb region (3215955–7261356) of KZ155846.1 chromosome; two SNPs (Record_7099 and Record_7097 loci) are distributed in the 250 b region (2481905–2482155) of KZ155908.1 chromosome; and the remaining three SNPs (Record_2315, Record_8964, and Record_396 loci) are distributed in KZ155852.1, KZ155945.1, and KZ155843.1 chromosomes, respectively. Linear regression analysis procedure was conducted on the 10 significant SNPs to narrow down the genomic region of chromosome KZ155846.1, verify the impact of the SNP marker on MW, improve the accuracy of the analysis model, and detect SNP–SNP combinations (SNP networks). In the construction of SNP networks—related to quantitative traits—several unique features of linear regression procedure were shown such as determining a leading marker within a gene related to a particular trait of interest, improving the correlation between the actual and predicted network values and predicting the average values of genotypes substitution in each SNPs [37]. Our linear regression analysis confirmed single-SNP associations and revealed three types of SNP networks related to MW (four SNP networks, three SNP networks, and two SNP networks). SNP network 4 trimmed the region of KZ155846.1 chromosome from ~4 Mb to ~56 Kb. As Record_1009 locus in SNP network 4 has an over dominant effect—which is rarely used in a practical selection program [37]—only two SNPs (SNPs Record_1111 and Record_1115 loci) are considered in the ~56 Kb region. Therefore, the SNP networks used in this study are a very powerful tool for optimizing candidate genes’ selection, thus facilitating the delineation of genetic variation that underlies complex traits such as growth performance [51]. Two SNP networks were established by linear regression model, which revealed two and three SNP networks for egg laying related SNPs in Yangzhou goose [16]. Multiple SNP networks using linear regression model analysis were established, which revealed two and three SNP networks for seven and five production traits in beef cattle, respectively [37].

The ten discovered genes in this study are novel and their association with growth in general and body weight of geese in particular has not yet been verified. Four out of the 10 genes are uncharacterized in geese genome. Despite there being some evidence indicating that the other six genes are associated with growth, their specific function has not yet been clearly defined in geese and further in-depth studies are needed. A nonsense mutation in the first exon of *Hmx1* gene causes a semi-lethal mutation in mouse called Dumbo, resulting in reducing body mass in survival mutants from ~3 days postpartum onwards with microphthalmic at puberty [52]. Eight SNPs of human *Lrrfip1* gene were found to be associated with abdominal fat, body fat percent, and C-reactive protein levels [53]. Micro RNA-132 blocks proliferation of vascular smooth muscle cells by inhibiting *Lrrfip1* expression [54]. Additionally, inhibition in cell growth and an increase in apoptosis caused by *GCF2/Lrrfip1* knockdown in human HepG2 cells [55]. *Ppp2r2c* plays various biological roles in dynamics and mobility of cytoskeleton [56], and control of cell growth and division [57]. A recent study revealed that *Ppp2r2c* is involved in the differentiation of bovine skeletal myoblast [58]. *Pdgfd* gene is a member of the platelet-derived growth factor family that is important for several types of connective tissue cells in terms of survival, function, and growth. *Pdgfd* was found to be potentially differentially regulated in obese rats’ skeletal muscle by essential polyunsaturated fatty acids [59]. A pair of *Uncx* and *Elfn1* genes are encoding transcription factors that coordinate growth and innervation of somatic muscles in zebrafish [60]. *Uchl1* may have a potential role in energy metabolism, as the evidence showed its unique role in muscle differentiation and lipid deposition [61]. *Uchl1* was the most downregulated gene involved in oxidative stress during both short- and long-term weight loss [62,63]. *Uchl1* was also identified as a differentially expressed gene in the longest dorsal and semi-membranous muscles in two Polish pig breeds differing in fat and meat qualities [64]. Based on these study results, we identified a novel mutation in the promoter region of *UCHL1* gene, which can alter transcriptional activity of *UCHL1* gene and affect the growth performance of male Yangzhou goslings [65].

In conclusion, the annotation analysis for the 10 genes and gene expression analysis emphasizes that these genes might be related directly or indirectly to growth performance. The mechanism by which the polymorphism in genes affects growth performance in geese has not been established. Whereas the polymorphisms evaluated in this study do not result in amino acid substitutions (all SNPs at down or upstream regions or in intron region of genes), these polymorphisms may be associated with other mutation(s) in other site(s) of the nucleotide sequence or other genes closely linked with these genes. This is the first case of discovery of novel genes that may be associated with growth performance in Yangzhou geese. The 10 SNPs can be recommended as potential genetic markers for growth traits in geese, but further verification studies are still needed to emphasize the current obtained results.

## 5. Conclusions 

SLAF sequencing with the BSA approach was applied for discovering and genotyping SNPs related to MW of male goslings. In general, 10 SNPs were confirmed to be associated with MW of males and only one SNP was associated with females. Ten genes located in five chromosomes were suggested to be associated with it. Interestingly, five genes are located at the ~4 Mb region of KZ155846.1 chromosome, which includes a ~56 Kb region containing three genes. We suggest this region with the 250 b region of KZ155908.1 chromosome to be related to the MW of male goslings, as shown by SNP network analysis. Moreover, all 10 SNPs can be potential target markers for marker-assisted selection to help identifying goslings with greater growth performance. Finally, our results confirmed that molecular methods such as SLAF sequencing with BSA can be used for determining molecular mechanisms underlying certain physiological cascade. To the best of our knowledge, the specific function of any of the 10 identified genes has not been clearly defined in geese and further in-depth studies are needed to explore the new functional role in MW of male goslings.

## Figures and Tables

**Table 1:** Statistics of sequencing data for each sample.

Sample ID *	Clean Reads	Total Reads	GC (%)	Q30 (%)	Total SNP	Heter. Ratio (%)	SLAF Number	Total Depth	Average Depth
HEBV	2.66 Gb	10,310,004	42.90	93.32	149.05	35.92	383.53	9,480,575	24.72
LEBV	2.73 Gb	10,571,785	42.86	93.21	149.05	37.72	384.63	9,726,388	25.29
Control	Rice	1,291,422	41.60	93.28					

* Sample ID: project sample number; HEBV, LEBV: high and low estimated breeding value groups, respectively; GC (%): the percentage of G and C bases in the total bases in the sequencing results; Q30 (%): the percentage of bases with a sequencing quality value greater than or equal to 30; Heter. ratio (%): single nucleotide polymorphism (SNP) heterozygosity in the sample; specific locus amplified fragment (SLAF) number: the number of SLAF tags contained in the sample; total depth: the total sequencing depth of the sample in the SLAF tags, that is, the total number of reads; and average depth: the average number of sequencing reads of the sample on each SLAF.

**Table 2:** The significant marketing weight-related SNPs including their genomic positions, ED, and *p*-values for HEBV and LEBV male and female goslings of first population.

SNP ID	SNP Type	Chr.*	Pos.	ED	Adjusted *p*-Value		Males		Females	
					FDR	Bonferroni	*p*	AIC	*p*	AIC
Record_1102	C/T	KZ155846.1	6778745	0.81	9.22 × 10^−^^33^	2.77 × 10^−^^32^	**7.87 × 10^−5^**	39.2	**0.021**	65.7
Record_1111	G/A	KZ155846.1	7205384	0.87	1.65 × 10^−18^	8.253 × 10^−^^18^	**3.64 × 10^−4^**	43.5	**0.033**	71.8
Record_2315	G/A	KZ155852.1	9238683	1.04	5.45 × 10^−^^16^	4.358 × 10^−^^15^	**1.06 × 10^−2^**	44.0	0.393	50.3
Record_1009	T/C	KZ155846.1	3215955	0.75	7.22 × 10^−^^16^	7.70 × 10^−^^15^	**3.93 × 10^−4^**	38.2	0.247	66.0
Record_1056	G/C	KZ155846.1	4966566	0.75	4.82 × 10^−^^15^	6.18 × 10^−^^14^	**2.34 × 10^−6^**	33.3	**0.027**	69.4
Record_7086	G/T	KZ155908.1	2312525	0.72	2.98 × 10^−^^8^	6.265 × 10^−^^7^	**4.45 × 10^−3^**	43.7	0.782	71.8
Record_1115	T/C	KZ155846.1	7261356	0.78	1.04 × 10^−7^	2.40 × 10^−^^6^	**3.98 × 10^−5^**	39.1	**0.006**	64.8
Record_7099	C/T	KZ155908.1	2482155	0.79	4.20 × 10^−^^7^	1.091 × 10^−^^5^	**8.44 × 10^−7^**	9.4	0.311	77.3
Record_7097	C/G	KZ155908.1	2481905	0.77	4.93 × 10^−^^7^	1.331 × 10^−^^5^	**8.79 × 10^−5^**	35.3	0.705	76.6
Record_8964	G/C	KZ155945.1	1664422	0.76	2.97 × 10^−^^6^	8.313 × 10^−^^5^	**3.22 × 10^−5^**	20.6	**0.009**	73.2
Record_1057	A/G	KZ155846.1	4997778	0.82	4.85 × 10^−^^6^	0.0001406	**3.59 × 10^−2^**	52.4	0.728	73.1
Record_396	T/C	KZ155843.1	11357652	0.71	6.74 × 10^−^^6^	0.0002155	**1.13 × 10^−6^**	-6.2	0.328	68.2
Record_11546	C/T	KZ156052.1	690770	1.07	2.26 × 10^−^^6^	0.0008542	**9.36 × 10^−3^**	41.7	0.093	71.4

* Chr: the chromosome number of GOOSE genomic; Pos: the position of SNP on chromosomes provided by the company; ED: Euclidean distance; FDR: false discovery rate; HEBV, LEBV: high and low estimated breeding value groups; *p*: probability (*p* < 0.05); and AIC: Akaike’s information criterion. Values of significant SNPs are in bold.

**Table 3:** The genotypic effects of the 10 SNPs of males in the first and second populations.

SNP Id *	Index	P1 Males				P2 Males			
		Values	SE	*p*	AIC	Values	SE	*p*	AIC
Record_1102	Additive	0.027	0.03	0.377	45.83	−0.114	0.05	**0.026**	21.87
	Dominance	−0.058	0.06	0.358	45.76	−0.211	0.08	**0.007**	19.46
	Recessive	−0.134	0.05	**0.005**	38.53	−0.062	0.08	0.450	26.38
Record_1111	Additive	−0.076	0.03	**0.007**	40.80	−0.124	0.05	**0.006**	17.10
	Dominance	−0.170	0.04	**0.000**	33.81	−0.198	0.10	0.051	20.85
	Recessive	−0.187	0.05	**0.000**	35.07	−0.148	0.06	**0.015**	18.62
Record_2315	Additive	0.265	0.10	**0.008**	51.18	−0.198	0.05	**0.000**	10.10
	Dominance	−0.185	0.16	0.258	57.04	−0.430	0.10	**0.000**	8.94
	Recessive	−0.453	0.10	**0.000**	38.18	−0.247	0.08	**0.002**	16.72
Record_1009	Additive	−0.067	0.03	**0.007**	38.64	−0.078	0.04	**0.045**	22.55
	Dominance	−0.151	0.04	**0.001**	34.33	−0.232	0.06	**0.000**	13.30
	Recessive	−0.120	0.05	**0.025**	40.90	0.006	0.07	0.930	26.71
Record_1056	Additive	−0.063	0.02	**0.010**	43.68	−0.166	0.05	**0.001**	7.77
	Dominance	−0.121	0.04	**0.005**	42.55	−0.258	0.10	**0.015**	13.35
	Recessive	−0.073	0.06	0.197	48.76	−0.187	0.06	**0.004**	11.04
Record_1115	Additive	0.003	0.03	0.928	47.53	−0.120	0.05	**0.011**	10.87
	Dominance	−0.212	0.07	**0.002**	38.14	−0.169	0.10	0.106	14.87
	Recessive	−0.167	0.04	**0.000**	32.02	−0.143	0.06	**0.021**	12.02
Record_7099	Additive	−0.118	0.02	**0.000**	−12.14	0.030	0.05	0.575	25.00
	Dominance	−0.214	0.04	**0.000**	−20.52	0.104	0.06	0.100	22.54
	Recessive	−0.139	0.04	**0.001**	−2.47	−0.245	0.12	0.053	21.47
Record_7097	Additive	−0.057	0.03	**0.014**	36.10	−0.091	0.05	**0.042**	18.62
	Dominance	−0.144	0.04	**0.001**	30.92	−0.154	0.06	**0.020**	17.26
	Recessive	−0.284	0.08	**0.000**	28.50	−0.070	0.08	0.404	22.17
Record_8964	Additive	−0.024	0.03	0.397	22.05	−0.096	0.04	**0.028**	22.53
	Dominance	−0.159	0.05	**0.002**	13.32	−0.076	0.08	0.317	26.48
	Recessive	−0.160	0.04	**0.000**	8.39	−0.176	0.07	**0.011**	20.76
Record_396	Additive	−0.056	0.04	0.138	−2.06	−0.131	0.04	**0.005**	1.98
	Dominance	−0.132	0.05	**0.010**	−6.63	−0.259	0.08	**0.001**	−0.52
	Recessive	−0.148	0.05	**0.002**	−9.21	−0.099	0.07	0.153	8.08

* SE: standard errors, *p*: probability (*p* < 0.05) and AIC: Akaike’s information criterion. Values of significant SNPs are in bold.

**Table 4:** Annotation of genes harboring SNPs associated with the marketing weight of male goslings.

SNP ID *	SNP	Acc. No.	Chr.	Pos. (bp)	Nearest Gene
Record_1102	C/T	NW_013185655.1	KZ155846.1	5835696	intron 4 *HMX1*
Record_1111	G/A	NW_013185655.1	KZ155846.1	5409310	29.5 Kb upstream *LOC106034756*
Record_2315	G/A	NW_013185684.1	KZ155852.1	2233138	10 Kb upstream *LRRFIP1*
Record_1009	T/C	NW_013185655.1	KZ155846.1	9373018	14.5 Kb upstream *LOC106035299*
Record_1056	G/C	NW_013185655.1	KZ155846.1	7629761	intron 1 *PPP2R2C*
Record_1115	T/C	NW_013185655.1	KZ155846.1	5353256	intron 1 *LOC106034755*
Record_7099	C/T	NW_013185939.1	KZ155908.1	659136	3.3 Kb downstream *UCHL1*
Record_7097	C/G	NW_013185939.1	KZ155908.1	659384	3 Kb downstream *UCHL1*
Record_8964	G/C	NW_013185681.1	KZ155945.1	769175	28.3 Kb downstream *LOC106034143*
				769175	41.2 Kb upstream *PDGFD*
Record_396	T/C	NW_013185677.1	KZ155843.1	7668615	24.6 Kb upstream *ELFN1*

* Acc No: accession number, Chr: the chromosome number of GOOSE genomic, and Pos: the position of SNP on gene.

## Data Availability

The raw sequence data have been submitted to the National Center for Biotechnology Information Short Read Archive with project accession of PRJNA718613; LEBV and HEBV SLAF-seq libraries were submitted with the accession numbers SRR14113841 and SRR14113842, respectively.

## Supplementary Materials

The following are available online at https://www.mdpi.com/article/10.3390/genes12081203/s1, Table S1: Information about candidate SNPs and their primers for goslings marketing weight, Table S2: Primer pairs of the candidate genes used for quantitative real-time PCR, Figure S1: The allele frequincies of the first population for males of LEBV (first row), males of HEBV (second row), females of LEBV (third row), and females of HEBV groups (forth row), Figure S2: Allelic and genotypic frequencies for males of both populations, whereas (first row) allelic frequency and (second row) genotypic frequency of P1 and (third row) allelic frequency and (forth row) genotypic frequency of P2.

## Abbreviations

AIC	Akaike’s Information Criterion
AS-PCR	Allele-specific polymerase chain reaction
BSA	Bulked segregant analysis
EBV	Estimated breeding value
ED	Euclidean distance
*ELFN1*	Extracellular leucine-rich repeat and fibronectin type III domain containing 1 gene
FDR	False discovery rate
GATK	Genome analysis toolkit
GC (%)	The percentage of G and C bases in the total bases in the sequencing results
GWASs	Genome-wide association studies
*HMX1*	H6 family homeobox 1 gene
*LRRFIP1*	LRR binding FLII interacting protein 1 gene
MW	Marketing weight (body weight at nine weeks of age)
P1	First population
P2	Second population
*PDGFD*	Platelet-derived growth factor D gene
*PPP2R2C*	Protein phosphatase 2 regulatory subunit B γ gene
Q30 (%)	The percentage of bases with a sequencing quality value greater than or equal to 30
SAMtools	Sequence alignment/map format
SLAF-seq	Specific locus amplified fragment sequencing
SNPs	Single nucleotide polymorphisms
SOAP	Short oligonucleotide alignment program
SSCP	Single-strand conformation polymorphism
ti/tv	Transition/transversion ratio
*UCHL1*	Ubiquitin C-terminal hydrolase L1 gene
