# Supplementary figures and images for: Identification and Validation of Marketing Weight-Related SNP Markers Using SLAF Sequencing in Male Yangzhou Geese
Source: Genes (Basel). 2021 Aug 3;12(8):1203. doi: 10.3390/genes12081203 (PMC8393582; doi:10.3390/genes12081203)

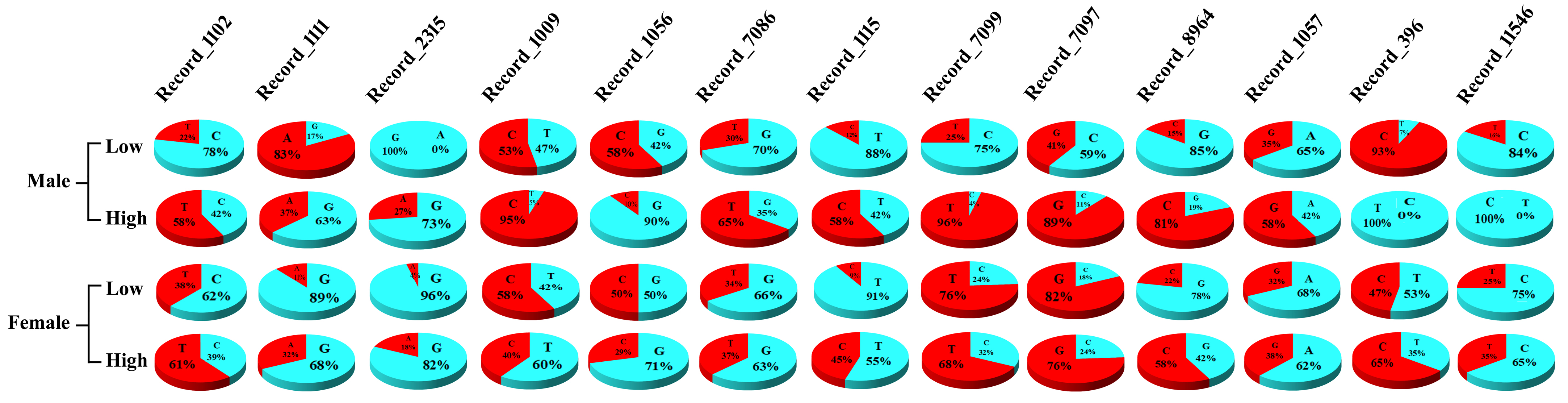

Supplement: Supplementary file 1 [file genes-12-01203-s001.zip › Supplementary/Figure S1. Allele frequencies of males and females in first population.jpg]

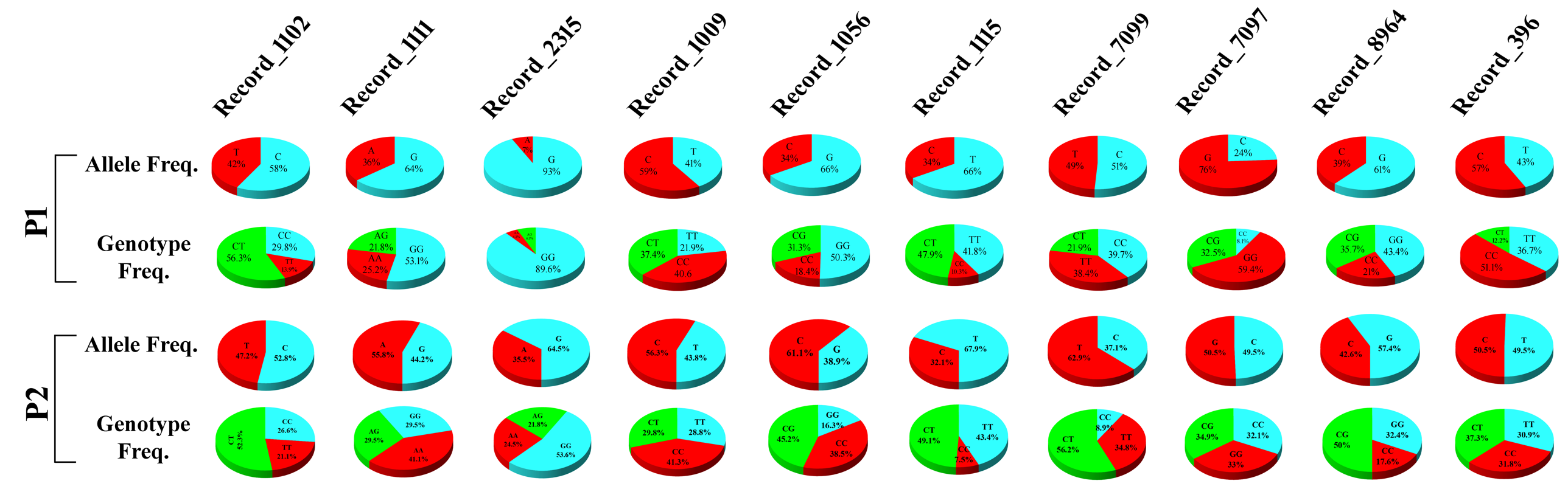

Supplement: Supplementary file 1 [file genes-12-01203-s001.zip › Supplementary/Figure S2. Allelec and genotypic frequencies for males of both populations.jpg]
